# Population substructure and space use of Foxe Basin polar bears

**DOI:** 10.1002/ece3.1571

**Published:** 2015-06-25

**Authors:** Vicki Sahanatien, Elizabeth Peacock, Andrew E Derocher

**Affiliations:** 1 Department of Biological Sciences, University of AlbertaEdmonton, Alberta, T6G 2E9, Canada; 2 Department of Environment, Government of NunavutIgloolik, Nunavut, X0A 0L0, Canada

**Keywords:** Movements, polar bear, satellite telemetry, sea ice, spatial ecology, *Ursus maritimus*

## Abstract

Climate change has been identified as a major driver of habitat change, particularly for sea ice-dependent species such as the polar bear (*Ursus maritimus*). Population structure and space use of polar bears have been challenging to quantify because of their circumpolar distribution and tendency to range over large areas. Knowledge of movement patterns, home range, and habitat is needed for conservation and management. This is the first study to examine the spatial ecology of polar bears in the Foxe Basin management unit of Nunavut, Canada. Foxe Basin is in the mid-Arctic, part of the seasonal sea ice ecoregion and it is being negatively affected by climate change. Our objectives were to examine intrapopulation spatial structure, to determine movement patterns, and to consider how polar bear movements may respond to changing sea ice habitat conditions. Hierarchical and fuzzy cluster analyses were used to assess intrapopulation spatial structure of geographic position system satellite-collared female polar bears. Seasonal and annual movement metrics (home range, movement rates, time on ice) and home-range fidelity (static and dynamic overlap) were compared to examine the influence of regional sea ice on movements. The polar bears were distributed in three spatial clusters, and there were differences in the movement metrics between clusters that may reflect sea ice habitat conditions. Within the clusters, bears moved independently of each other. Annual and seasonal home-range fidelity was observed, and the bears used two movement patterns: on-ice range residency and annual migration. We predict that home-range fidelity may decline as the spatial and temporal predictability of sea ice changes. These new findings also provide baseline information for managing and monitoring this polar bear population.

## Introduction

William Henry Burt was an early proponent of an integrated approach to wildlife management conservation, when he identified the need to understand a species’ behavior and spatial patterns (Burt 1943; Lima and Zollner 1996). Today, knowledge of individual movements, home range, and habitat is considered basic requirements of species conservation and management (Mueller et al. 2011; Nagy et al. 2011). Technology now allows collection of high frequency, high resolution geographic position system (GPS) location, activity, and environmental information that can be used to understand behavior and habitat use. Such data make it possible to study wide ranging species in remote regions, such as the Arctic, using satellites to relay next to real time information (Kie et al. 2010).

Population structure is dynamic and can be distinguished at a variety of temporal scales ranging from the movement of species in geological time to shorter times scale and ecologically important events such as dispersal and migration (Greenwood 1980; Mueller and Fagan 2008; Revilla and Wiegand 2008). Changing habitats and resource distribution can alter population size, dispersal patterns, and distribution (Parmesan and Yohe 2003; O’Corry-Crowe 2008). Climate change has been identified as a major driver of habitat change (Post et al. 2009; Wassmann et al. 2011). Within this context, Arctic habitats have experienced greater warming than lower latitudes (Trenberth et al. 2007; IPCC 2013). Of particular concern are species of large Arctic marine mammals that have small population sizes, slow reproductive rates, and specialized life histories that make them vulnerable to climate change (Stirling and Derocher 1993; Tynan and DeMaster 1997; Laidre et al. 2008; Gilg et al. 2012). Polar bears (*Ursus maritimus*) are one such vulnerable species, due to their high trophic level, specialized diet, and reliance on the distribution of sea ice habitat for foraging (Derocher et al. 2004; Stirling and Derocher 2012).

Polar bears are distributed throughout the circumpolar Arctic in close association with the distribution of sea ice (DeMaster and Stirling 1981). Historically, their widespread distribution led Pedersen (1945) to speculate that polar bears consisted of a single large intermingling population. In the 1970s, when marked bears were recaptured or harvested, population spatial structure and regional fidelity were revealed (Lentfer 1973; Stirling et al. 1977). The first statistical assessment of polar bear spatial organization using satellite telemetry data identified the existence of geographically constrained populations (Bethke et al. 1995). As more detailed movement datasets accumulated, population delineations were revised (Taylor et al. 2001; Mauritzen et al. 2002; Amstrup et al. 2004). The IUCN/SSC Polar Bear Specialist Group has collated circumpolar input to delineate 19 populations based on a combination of telemetry data, geographic barriers, genetics, fidelity to summer ranges, and tag returns from hunters (Obbard et al. 2010); these populations serve as a basis for conservation, management, and harvest (Vongraven et al. 2012). Genetic analysis of polar bears has identified four broad groupings with subgroups of varying levels of distinction (Paetkau et al. 1999; Peacock et al. 2015). The population structure arises from spatial and temporal fidelity (Mauritzen et al. 2001; Lone et al. 2012). For example, pregnant females show fidelity to denning areas, return with their offspring and thereby may imprint travel routes and spatial information on their young (Derocher and Stirling 1990; Ramsay and Stirling 1990). The benefits of site fidelity in a species with such prodigious abilities to move long distances (Taylor and Lee 1995; Paetkau et al. 1999) remain poorly understood.

Polar bear sea ice habitat has four broad ecoregions (divergent, convergent, archipelago, and seasonal) based on-ice composition, circulation, duration, and how bears respond to sea ice dynamics (Amstrup et al. 2008). Much of our understanding of polar bear sea ice spatial ecology has come from studies in the high Arctic where there is a mixture of multiyear and annual sea ice and polar bears have access to sea ice habitat year round (Amstrup et al. 2000; Ferguson et al. 2001; Mauritzen et al. 2002). These high Arctic regions are part of the divergent, convergent, and archipelago ecoregions (Amstrup et al. 2008). The fourth zone, the seasonal ice ecoregion, includes five populations (Southern Hudson Bay, Western Hudson Bay, Foxe Basin, Davis Strait and Baffin Bay) where polar bears must retreat to land each summer when sea ice melts. Bear movements have been examined in HB (Parks et al. 2006; Obbard and Middel 2012), Baffin Bay (Ferguson et al. 1997, 1999; Taylor et al. 2001), and Davis Strait (Taylor et al. 2001). Delayed freeze-up and earlier break-up, correlated with increasing surface air temperatures, has reduced the duration of ice cover in the seasonal ice region (Moore 2006; Stirling and Parkinson 2006; Hochheim and Barber 2010; Galbraith and Larouche 2011; Sahanatien and Derocher 2012) with negative consequences for polar bear population status and persistence (Regehr et al. 2007; Amstrup et al. 2008; Rode et al. 2012; Castro de la Guardia et al. 2013).

This is the first study to investigate the spatial ecology of female polar bears in the seasonal sea ice ecoregion of Foxe Basin in Nunavut, Canada, using satellite telemetry. Our objectives were to examine intrapopulation spatial structure, to determine movement patterns or strategies, to consider how polar bear movement behavior may respond to changing sea ice habitat conditions, and to provide a baseline of information for management and monitoring.

## Methods

### Study area

**Figure 1:**
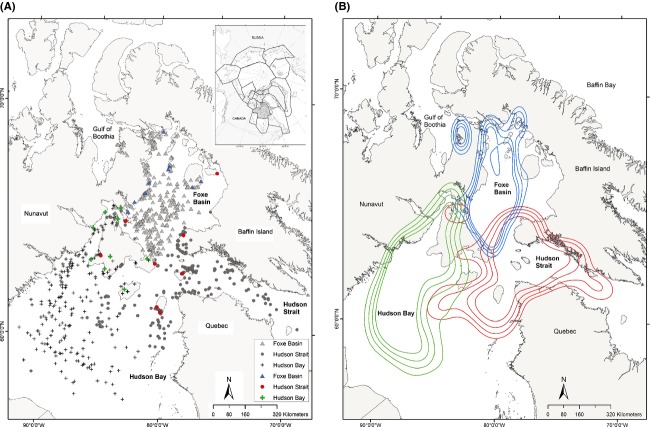
(A) Study area, with female polar bear geographic position system satellite telemetry movement locations (black symbols) and capture locations (color symbols), coded according to subpopulation cluster assignment, Foxe Basin (FB), Nunavut, Canada, October–March 2007–2011. (B) Kernel distribution (50%, 60%, 70%, and 80%) contours of Foxe Basin (blue), Hudson Strait (red), and Hudson Bay (green) subpopulation clusters in Foxe Basin, Nunavut, Canada, October–March 2007–2011.

Polar bears were caught and collared on land in the Foxe Basin polar bear population management unit, which includes Foxe Basin, northern Hudson Bay and western Hudson Strait in Nunavut, Canada (Fig.1). The collars were distributed across the region to insure spatial coverage for management unit delineation and characterizing intrapopulation spatial structure (this analysis). The polar bears of this population were historically hunted by Inuit and others (e.g., whalers); hunting continues and has been managed by a population specific quota and nonquota hunter restrictions since the early 1970s (Stirling and Smith 1974).

The study area was delineated by the locations of collared bears covering approximately 800,000 km^2^ and included Foxe Basin, Hudson Strait central and northern Hudson Bay, and eastern Gulf of Boothia (Fig.1). The area extended approximately 1300 km from south to north and 1400 km from east to west. Hudson Bay is the largest water body (840,625 km^2^), with Hudson Strait (196,875 km^2^) and Foxe Basin (203,750 km^2^) being similar in size. All three areas are shallow (predominantly <200 m deep), productive seas that undergo an annual sea ice cycle from ice free to almost total ice cover (Prinsenberg 1986b). Freeze-up begins in October and is complete in late December or January; break-up begins in May and continues into August (Saucier et al. 2004; Fequet et al. 2011). The timing of freeze-up was average in 2007–2008, but in 2008–2009, 2009–2010, and 2010–2011 above normal air temperatures slowed the growth of sea ice resulting in thinner ice (Canadian Ice Service 2008, 2009, 2010, 2011). In 2010–2011, development of average winter ice concentration and extent was delayed by 4 weeks in Foxe Basin, 6 weeks in Hudson Bay, and 8 weeks in Hudson Strait.

Ocean currents and coastline configuration play important roles in the development and distribution of sea ice. Foxe Basin and Hudson Bay have cyclonic circulation that results in active, centrally circulating floe ice bordered by a strip of stable landfast ice (Prinsenberg 1986a; Fequet et al. 2011). Hudson Strait also has narrow strips of landfast ice adjacent to active floe ice that moves linearly with the dominant west to east current. Hudson Strait is the outflow of Hudson Bay and Foxe Basin to the Atlantic Ocean. The coastline morphology of Hudson Bay is smooth and regular with few offshore islands; in contrast, Foxe Basin and Hudson Strait coastlines are more complex with many islands. There is a diversity of polar bear prey species including: ringed seals (*Pusa hispida*) (the main prey species), bearded seals (*Erignathus barbatus*)*,* harbour seals (*Phoca vitulina*), harp seals (*Pagophilus groenlandicus*), walrus (*Odobenus rosmarus*), bowhead (*Balaena mysticetus*), narwhal (*Monodon monoceros*), and beluga (*Delphinapterus leucas*) (Sergeant 1986; Smith and Sjare 1990; Stewart and Lockhart 2005; Schliebe et al. 2008; Thiemann et al. 2008).

### Capture and deployment of satellite collars

Bears were immobilized using tiletamine hydrochloride and zolazepam hydrochloride (Telazol; Fort Dodge Laboratories, Fort Dodge, IA) by remote injection using a dart delivered from a helicopter. All bears were caught on land, during the ice-free season, following standard capture and handling methods (Stirling et al. 1989). Animal handling protocols were approved by the University of Alberta Animal Care and Use Committee for Biosciences.

Global positioning system satellite collars (Telonics, Inc., Mesa, AZ) linked to Argos satellites (CLS America, Lanham, MD) were deployed on 45 adult female polar bears with cubs-of-the-year, yearlings, and 2-year olds, as well as, two females without offspring in August–October, 2007–2009.

Location data were collected at 3- or 4-h intervals. We used GPS quality location data (accuracy <10 m) with the exception of two bears whose collars provided only Doppler shift quality locations for part of the year, of which we used classes 1, 2, and 3 (accurate to <1 km). Daily locations at 13:00 GMT or nearest recorded values were used for the cluster analysis and overlap analyses, and all locations were used to calculate movement rates and home-range size.

We defined five seasons based on sea ice dynamics, ice concentration, and ringed seal life history, similar to those used by Ferguson et al. (2001) and Parks et al. (2006). The seasons were as follows: ice-free (minimum to no sea ice and bears were on land), freeze-up (when a bear moved onto the ice until December 31), winter (when sea ice concentration was 90–100%, 01 January–31 March), spring (when ringed seals pup and molt, 01 April–31 May), and break-up (when the sea ice melts and independent seal pups are available, 01 June, until ice-free conditions dominate and the date that a bear returned to land).

If a bear entered a maternity or temporary den, the locations were excluded in the movement rate calculations but were included in the home range and cluster analyses. Denning was identified when a bear stopped moving for several weeks or months, and renewed movement was not attributed to sea ice movement. All suspected dens were on land.

### Spatial structure of movements

We used hierarchical cluster analysis (Bethke et al. 1995; Schaefer et al. 2001; Nagy et al. 2011) to investigate whether there was spatial structure and regional affinities in the movements of the Foxe Basin polar bear population using location data from the on-ice period. This data subset focuses on movement responses to the sea ice habitat where polar bears obtain most of their annual energy stores while foraging and includes their distribution during the spring mating season. Weekly median locations (latitude, longitude) were calculated for 27 bears for October–March, representing 35 ice years of movement information. The median location values were converted to metric x, y coordinates using Hawth’s Tools (Beyer 2004). SPSS v19 (IBM, Somers, NY) was used for the agglomerative hierarchical cluster analysis, using Ward’s linkage (Bethke et al. 1995) which minimizes the within-cluster variance versus the between-cluster variance (Ludwig and Reynolds 1988).We used STATA 10 (STATCORP, College Station, TX) to calculate the posthierarchical clustering Duda–Hart pseudo-*t*-test (Rabe-Hesketh and Everett 2007) to identify the optimum number of distinct groups. FUZME v3.5 (Minasny and McBratney 2002) was used to apply fuzzy c-means clustering as a third analytical approach to examine the optimum number of clusters and the assignment of bears to each cluster following Schaefer et al. (2001) and Nagy et al. (2011). We used a relatively low level of fuzziness or “hard” classification (*m* = 1.5) and the diagonal metric given the dimensions of the study area. FUZME has been applied to determine caribou (*Rangifer tarandus*) herd membership (Schaefer et al. 2001; Nagy et al. 2011; Schaefer and Mahoney 2013). To test for independent or coordinated movements within the FB -collared bears, we calculated the fuzziness performance index (FPI) and modified partition entropy index (MPE) using FUZME. The clusters were mapped using kernel density distributions calculated using Home Range Tools for ArcGIS® v1.1 (Rodgers et al. 2007).

### Spatial and movement metrics

To allow comparison with previous studies, we calculated minimum (100%) convex polygon (MCP) home ranges. Annual and seasonal MCPs were calculated using Hawth’s Tools (Beyer 2004) in ArcMap v9.3.1 (Environmental Systems Research Institute, Inc., Redlands, CA) for bears with >9 months of location data using all available locations. Most bears were collared in September and October, thus 9 months of data included most of the on-ice period and did not influence individual annual MCP area, but break-up season MCPs were not calculated for bears with truncated location data.

Movement rates and Euclidean distances between locations were calculated using Hawth’s Tools in ArcMap v9.3.1 (Environmental Systems Research Institute, Inc.). All available locations were used to calculate monthly movement rates and for each month a bear had to have ≥15 days of location data to be included. The total on-ice time (days) was calculated for each bear as the date on ice at freeze-up to the date on land the following year.

To compare differences between the movement metrics, we used a one-way analyses of variance (ANOVA) and Tukey’s HSD post hoc test for pair-wise comparisons. ANOVA was used to test for significant trends in monthly and seasonal movement metrics. All statistical tests and comparisons used *α *= 0.05 and were performed using SPSS. Means are presented with ± 1 standard error (SE).

### Home range fidelity

Home range fidelity was measured by calculating individual interannual seasonal and annual home-range overlap. Nine females from Foxe Basin and Hudson Strait had sufficient data over two consecutive years to be included in the overlap calculations. We used both static and dynamic overlap (Powell 2000), which were calculated using Ranges8 v2.8 (Anatrack Ltd., Wareham, UK). Static overlap describes the spatial overlap of home ranges and was used to quantify the overall repeated use of available habitat. Static overlap (0–100%) was calculated by measuring the percentage of home range overlap of Year 1 on Year 2 and Year 2 on Year 1. Dynamic overlap, also called interactive overlap, incorporates time and space by analyzing the relationship between pairs of locations (Powell 2000). In our study, paired daily locations of individual bears recorded 1 year apart were used to assess spatiotemporal home range fidelity. We calculated Jacobs’ index in Ranges8 to measure dynamic overlap between years for each bear. The observed and possible distances (1000 random locations) between paired locations were compared to calculate Jacobs’ Index, which ranges from −1 (avoidance and no fidelity in this analysis) to +1 (attraction and complete fidelity in time and space), with 0 indicating independence of locations (Kenward et al. 2008). Jacobs’ index has been used to measure dynamic overlap and sociality between individuals, sexes, and species (Zalewski and Jedrzejewka 2006; Schmidt et al. 2009; Mattisson et al. 2011).

## Results

### Spatial structure of movements

**Figure 2:**
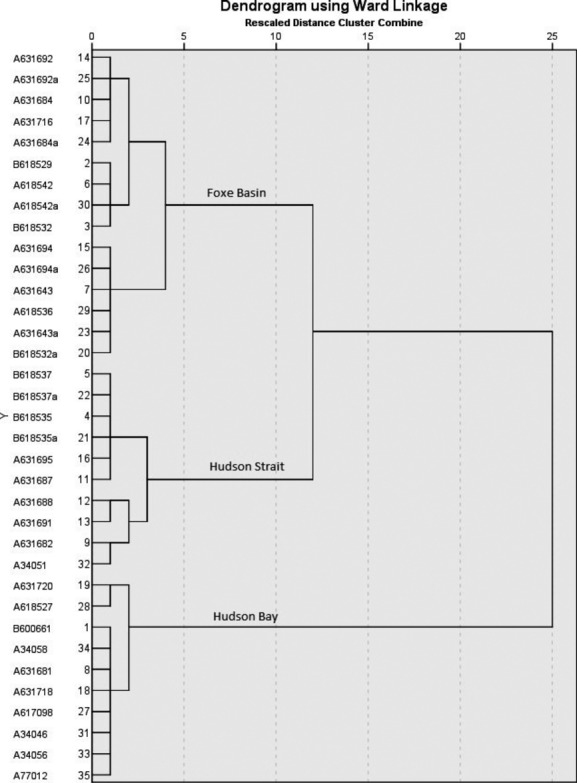
Dendrogram showing three geographic clusters of Foxe Basin female polar bears (*n* = 35) using geographic position system satellite telemetry median weekly locations for the on-ice period, October–March, 2007–2011.

**Figure 3:**
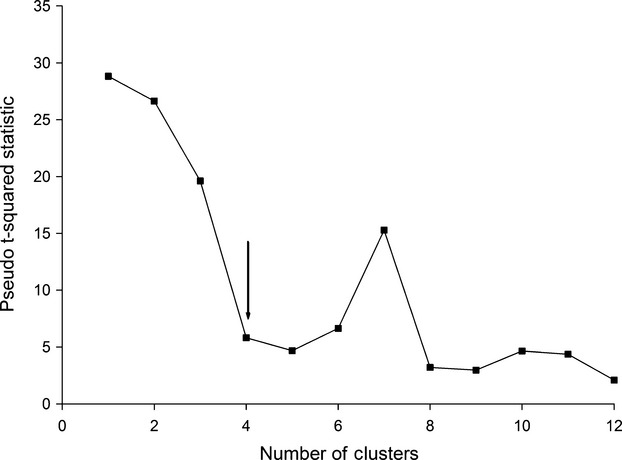
Duda-Hart pseudo t-squared test statistic graphed with the inflection point (arrow) indicating the potential optimum number of spatial clusters of female polar bears (n = 35) in the Foxe Basin population using median weekly on-ice GPS satellite telemetry locations (October - March, 2007-2011).

Three spatial clusters were identified within the Foxe Basin population that broadly coincided with the main water bodies: Foxe Basin (FB), Hudson Strait (HS) and Hudson Bay (HB) (Fig.1A,B). The agglomerative hierarchical cluster analysis separated out the HB cluster at the first-order level and the FB and HS clusters at the second-order level (Fig.2). A fourth cluster may be present based on the inflection point of the Duda–Hart pseudo-*t*-squared test (Fig.3). The fuzzy cluster results showed 2–4 possible geographic clusters (see Fig. S1 and Table S1 in Supporting information). At the third order, the hierarchical cluster analyses results split the FB cluster, but the fuzzy cluster analysis results (see Fig. S1 and Table S1) split the HB cluster. Because there was disagreement on membership of bears in a fourth cluster, but agreement between three analytical approaches on the composition of the three clusters, we based our subsequent comparisons on three clusters. The plotted fuzziness performance (FPI) and modified partition entropy (MPE) indices reached minima at zero for most values of the fuzziness weighting exponent (*m*), showing that each bear was spatially independent (see Fig. S1 and Table S1).

### Spatial and movement metrics

The mean annual MCP home range area was 115,918 ± 15,382 km^2^ and varied from 19,633 km^2^ to 401,351 km^2^. The mean annual home range sizes differed between clusters within the FB population (*F*_2,26_ = 6.15, *P* = 0.006, Table1). The FB cluster mean annual home range was smaller than in HB (Tukey’s HSD, *P* = 0.006). There were also differences in the seasonal home range sizes. The freeze-up home ranges differed among clusters (*F*_2,29_ = 9.35, *P* = 0.001), with those in FB and HS smaller than in HB (Tukey’s HSD, *P* = 0.001; Tukey’s HSD, *P* = 0.003). Winter home ranges also differed among clusters (*F*_2,31_ = 3.24, *P* = 0.05), with FB being smaller than HS and HB (Tukey’s HSD, *P* = 0.05).

**Table 1:** Mean annual and seasonal home range (minimum convex polygon) sizes (km^2^) of geographic position system satellite-collared female polar bears in Foxe Basin (FB), Hudson Strait (HS), and Hudson Bay (HB), Canada (2007–2011).

Region	Annual	Standard error (SE)	*n*	Freeze-up	SE	*n*	Winter	SE	*n*	Spring	SE	*n*	Break-up	SE	*n*
FB	**59137**	9093	11	**21187**	4223	13	**13465**	2758	14	11863	2995	13	18055	4393	10
HS	**132760**	25493	8	**21415**	8008	9	**27767**	8407	10	22995	5811	8	28588	6734	6
HB	**164904**	30714	10	**53371**	6305	10	**37139**	9745	10	24235	8541	8	50791	22515	3

Bolded values are significantly different (one-way analyses of variance, *P* < 0.05)

**Figure 4:**
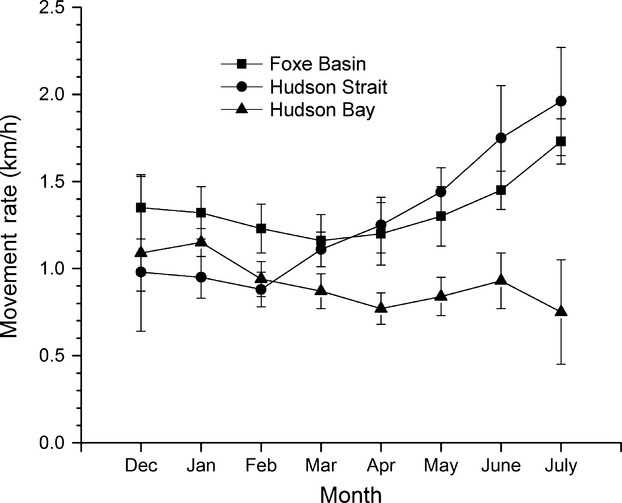
Mean monthly on-ice movement rates (km/h) of geographic position system satellite-collared female polar bears in Foxe Basin, Hudson Strait, and Hudson Bay, Canada (2007–2011).

Mean seasonal movement rates ranged from 0.9 km/h during freeze-up and winter to 1.8 km/h during break-up (Table2). Regional movement rates significantly differed only during spring (*F*_2,26_ = 3.57, *P* = 0.04) when HB (0.8 km/h) bears moved slower than HS (1.3 km/h) and FB (1.2 km/h) bears. Mean monthly on-ice movement rates from December to July declined in HB (*F*_1,6_ = 10.16, *P* = 0.02), increased in HS (*F*_1,6_ = 41.09, *P* < 0.001), and there was no trend in FB (*F*_1,6_ = 2.92, *P* = 0.14) (Fig.4).

**Table 2:** Mean seasonal movement rates (km/h) of geographic position system satellite-collared female polar bears in Foxe Basin (FB), Hudson Strait (HS), and Hudson Bay (HB), Canada (2007–2011).

Region	Freeze-up	Standard error (SE)	*n*	Winter	SE	*n*	Spring	SE	*n*	Break-up	SE	*n*
FB	1.3	0.1	17	1.1	0.1	16	**1.2**	0.1	11	1.5	0.1	10
HS	0.9	0.1	12	1.1	0.1	13	**1.3**	0.1	8	1.8	0.3	5
HB	1.2	0.1	16	0.9	0.1	13	**0.8**	0.1	10	1.1	0.1	4

Bolded values are significantly different (one-way analyses of variance, *P* < 0.05).

The mean number of days on the sea ice differed among the regions (*F*_2,12_ = 8.83, *P* = 0.004). FB bears were on the ice 31 days longer (Tukey’s HSD, *P* = 0.03) than bears in HS and 56 days longer (Tukey’s HSD, *P* = 0.007) than bears in HB (Table3). The mean date that bears moved on to the sea ice differed among regions (*F*_2,51_ = 15.62, *P* < 0.001). Polar bears moved onto the sea ice in FB the earliest (Tukey’s HSD, *P* ≤ 0.001), and HS the latest (Tukey’s HSD, *P* = 0.001). The mean date that bears left the sea ice for land also differed among regions (*F*_2,14_ = 13.98, *P* < 0.001) and FB bears left the ice for land latest (Tukey’s HSD, *P* ≤ 0.001) and HB bears earliest (Tukey’s HSD, *P* = 0.003).

**Table 3:** Time spent on the sea ice by female polar bears in Foxe Basin (FB), Hudson Strait (HS), and Hudson Bay (HB), Canada (2007–2011).

Region	Mean days on-ice	Standard error (SE)	*n*	Mean ordinal date on-ice	SE	*n*	Mean ordinal date off-ice	SE	*n*
FB	**294**	6	7	**306 (November 01)**	3	33	**238 (August 25)**	3	8
HS	**263**	10	6	**336 (December 01)**	6	15	**227 (August 14)**	6	7
HB	**238**	4	2	**313 (November 08)**	2	16	**187 (July 05)**	1	2

Bolded values are significantly different (one-way analyses of variance, *P* < 0.05).

### Home range fidelity

**Figure 5:**
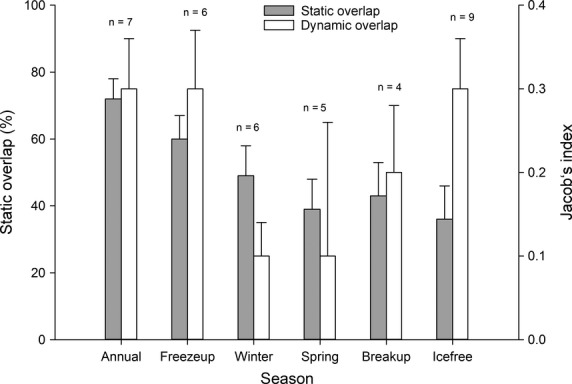
Seasonal and annual static minimum (100%) convex polygon overlap and dynamic home-range overlap of female polar bears in Foxe Basin, Hudson Strait, and Hudson Bay, Canada (2007–2011).

Female polar bears demonstrated annual and seasonal home range fidelity in FB and HS. It was not possible to calculate HB bears’ annual home range fidelity due to lack of data. There was a high level of annual and seasonal static overlap of individual polar bear home ranges (*n* = 9). The mean annual static overlap was 72 ± 6% (range 24–92%). The mean seasonal static home range overlap ranged from 36 ± 10% during the ice-free season to 60 ± 7% during freeze-up (Fig.5). Over the year, the individual seasonal overlap values ranged from 0% to 99%.

Annual home range dynamic (temporal and spatial) overlap was positive with mean Jacobs’ index of 0.3 ± 0.06 (range 0.2–0.6) (Fig.5). A positive Jacob’s index means that individual bears are near the same geographic locations at the same times of year. Seasonal home range dynamic overlap was variable but generally positive, with individual bear Jacobs’ index values ranging from −0.2 to 0.7. Bears had higher mean seasonal Jacob’s indices during freeze-up, break-up, and ice-free seasons. The pattern of static and dynamic home range overlap was similar except during winter and spring.

## Discussion

Within the Foxe Basin population, female polar bear locations on the seasonal sea ice were distributed in three clusters and each cluster generally corresponded with the main marine regions. There were differences in the movement metrics between clusters. At the individual level, the bears showed annual and seasonal fidelity to their home ranges and moved independently of each other on the sea ice.

Intrapopulation spatial structure has been observed in other polar bear populations. Cluster analyses in the Barents Sea and Kara Sea populations found that the spatial clusters were related to sea ice habitat use: One cluster used seasonal fast ice in the near shore, and the other used multiyear drift ice off shore (Mauritzen et al. 2002). In Davis Strait, population and genetic cluster analyses indicated two geographic groups: northern and southern Davis Strait that corresponded to the main coastal summer ice-free retreat areas (Taylor et al. 2001; Peacock et al. 2015). The genetic clusters also differed in prey (Iverson et al. 2006), birth rates, and survival rates (Peacock et al. 2013). In the Beaufort Sea, utilization distribution analysis revealed that “homebody” bears, those with small annual ranges, formed intra-population clusters (Amstrup et al. 2004). In southern Hudson Bay, seasonal utilization distributions revealed that polar bears were distributed in two spatial groups: James Bay and southern Hudson Bay (Obbard and Middel 2012), which was supported by genetic analysis (Peacock et al. 2015), suggesting breeding season substructure.

In the 1980s, based on marks returned by Inuit hunters, it was hypothesized that there were two geographic units of bears in Foxe Basin (Stirling and Ramsay 1986): one group in the north (Foxe Basin and Hudson Strait) and the other in the south (northern Hudson Bay). Our findings, however, provide empirical evidence based on the distribution and movements of polar bears. Hierarchical cluster analysis of animal movement data can be challenging to interpret as different approaches can yield varying or conflicting results (Bethke et al. 1995; Mauritzen et al. 2002; Schaefer and Wilson 2002; Amstrup et al. 2004; Nagy et al. 2011). Determining the number of clusters can be particularly difficult in species with a continuous distribution and individual movement patterns. By taking a parsimonious approach, applying knowledge of behavior and ecological needs and considering the movement metrics associated with the clusters, it is reasonable to conclude that the FB population is comprised of three spatial clusters.

Because the sea ice habitat is similar throughout our study area, seasonal ice with active pack ice and a small fraction of landfast ice (http://iceweb1.cis.ec.gc.ca/), the clusters were likely unrelated to differential use of sea ice habitats. Further, there were no apparent barriers (e.g., mountain ranges, vast open water) to polar bear movement, and both prey and denning habitats were widely available over the region. Polar bears were capable of moving across the entire study area but they did not, instead they exhibited regional clusters that adhered to marine regions of Foxe Basin, Hudson Strait, and Hudson Bay. The clusters arose from individual home-range fidelity and may be a product of learned behavior that develops in a predictable and resource-rich environment (Mueller and Fagan 2008). The clusters were also affected by ecological differences created by sea ice dynamics.

Dynamic sea ice habitats were thought to have unpredictable resource distributions that influenced polar bears’ movements, and thus, discrete home ranges were unexpected and female distribution assumed to vary over time (Ramsay and Stirling 1986, 1988). We agree that polar bear movements are coupled with sea ice structure and distribution, but the labile ice may not be as unpredictable as previously thought. With the advantage of modern satellite imagery and ice maps, we observed that sea ice is dynamic, but broadly predictable at larger spatial and longer temporal scales in Foxe Basin. This predictability makes it possible for polar bears to have home ranges in the traditional sense, where bears move repeatedly through a definable space over months and years (Powell 2000), as demonstrated by our population overlap metrics. If sea ice was an unpredictable habitat, it would be conducive to nomadism (Mueller and Fagan 2008) which polar bears do not show.

Home-range fidelity provides familiarity with the distribution of resources (Zalewski and Jedrzejewka 2006; Wolf et al. 2009; Spencer 2012) and may be necessary for the highly seasonal feeding behavior of polar bears. The on-land fasting period in Foxe Basin is 2.4–4 months long and similar to other seasonal ice populations (Parks et al. 2006; Cherry et al. 2013). The existence of fidelity to summer retreat areas, spring feeding and breeding areas in Foxe Basin was questioned because ice persists there longer than in Hudson Bay (Stirling and Ramsay 1986). While our sample size was small and included only two consecutive years, we provide evidence that Foxe Basin bears had fidelity to retreat and spring feeding areas, and likely breeding areas. This fidelity is concordant with the accumulating observations of repeated use of sites and area fidelity on annual and seasonal time frames in seasonal and multiyear ice ecoregions (Schweinsburg et al. 1982; Derocher and Stirling 1990; Born et al. 1997; Amstrup et al. 2000; Mauritzen et al. 2001; Wiig et al. 2003; Lone et al. 2012).

Differences in polar bear space use metrics (e.g., home range size, movement rate) have been related to foraging strategies and ultimately with the structure and quality of sea ice habitat. In the Canadian Arctic and Barents Sea, female polar bears using pelagic or active pack ice habitat had larger annual ranges than the near-shore or landfast ice bears (Ferguson et al. 1999; Mauritzen et al. 2001). Ferguson et al. (1999) proposed that home range size reflected the predictability of the environment and prey; in pelagic or active pack ice, prey distribution may be less predictable than on landfast ice and require more effort to find. Home range size can be influenced by habitat productivity or food availability with high productivity yielding smaller home range size (McLoughlin and Ferguson 2000; Moyer et al. 2007; Edwards et al. 2013). Such findings are predicted by the resource dispersion hypothesis for territorial and social carnivores (Macdonald 1983; Newsome et al. 2013). The cluster level differences in movement metrics of the Foxe Basin population may reflect the habitat conditions experienced by the bears. Like most movement studies of polar bears, we did not have quantitative information on prey distribution and density to evaluate habitat quality. Nonetheless, our results on home range size coupled with days on-ice suggest that habitat quality in Hudson Bay is lower than in Foxe Basin and Hudson Strait. The HB cluster of bears had the largest home-range size, fewest days on-ice, and earliest off-ice date. The bears in the FB cluster had the smallest home ranges, greatest number of days on-ice and latest off-ice date. Based on Ferguson et al. (1999), we would have expected the movement rate to be highest in the HB cluster, but it was the lowest, suggesting that prey availability is at a level such that conserving energy may be a factor. Hudson Bay has lower phytoplankton production and biomass (Ferland et al. 2011; Cyr and Larouche 2014) and lower zooplankton biomass than Hudson Strait and Foxe Basin (Estrada et al. 2012) to support polar bears and their prey (Hobson and Welch 1992; Bluhm and Gradinger 2008).

Resource distribution and landscape structure are major drivers of individual movement behavior and mechanisms (spatial memory, oriented, and nonoriented) that results in emergent population level patterns and strategies (range residency, migration, and nomadism) (Mueller and Fagan 2008). Mueller and Fagan’s conceptual framework of temporal and spatial resources gradients with levels of heterogeneity and predictability in dynamic environments can be extended to the sea icescapes used by polar bears. In Foxe Basin, female polar bears during the sea ice season would be considered range residency strategists as we observed home-range fidelity, which reflects spatial memory, annual and seasonal temporal predictability of sea ice habitat, and the predominant active floe ice that creates fine-scale heterogeneity in the distribution of prey habitat. Similar to the polar bears of the adjacent Western Hudson Bay, Foxe Basin bears must move from sea ice to land for the ice-free season and the movement on shore has been described as migratory (Cherry et al. 2013). Our observations suggest that Foxe Basin polar bears use both seasonal on-ice range residency and annual migration; the use of dual movement patterns has been described in other species (Mueller and Fagan 2008; Mueller et al. 2011). The switch between strategies is likely caused by fine-scale changes in the physical environment experienced by the bears. When sea ice drops below a threshold concentration, the bears move to land (Cherry et al. 2013).

Within the context of climate change, understanding polar bear movement patterns are important as a means to anticipate their potential behavioral plasticity for responding to changes in sea ice habitat phenology, distribution, and loss. Research (Ferguson et al. 2000; Mauritzen et al. 2001) suggests that polar bears have behavioral plasticity on short temporal scales (day to day, week to week, even month to month) and can exploit most sea ice habitat types (multiyear, annual, fast, and drifting pack ice), but we do not know how flexible they are on annual and decadal time scales. Can individual bears switch movement strategies as environment changes? Ferguson et al. (1999) thought that polar bears could switch from a pelagic to a fast ice strategy, which implies less reliance on home-range fidelity. In contrast, Mauritzen et al. (2001) proposed that polar bears had a single strategy and stayed with it over their lifetime and concluded that dynamic sea ice is a predictable habitat at relevant spatial and temporal scales. In Foxe Basin, ice seasons are changing and there has been increasing fragmentation of sea ice habitat (Stirling and Parkinson 2006; Sahanatien and Derocher 2012). If the spatial and temporal predictability of resources and sea ice habitat declines, we predict home-range fidelity may decline.

Polar bear populations can be affected by the dual stresses of habitat change and hunting (Lentfer 1983; Peacock et al. 2011). Both factors will continue to influence polar bear populations, particularly in seasonal sea ice regions. The Foxe Basin polar bear population allowable harvest is set for the entire management unit, but the existence of three clusters suggests that management should consider population substructure. Changing ice conditions and the differences in the ice-free period suggests that the demographics of the population may vary geographically. To date, demographic analyses have been conducted on a population basis, but in areas with complex habitat structure, it is important to consider regional demographics (Peacock et al. 2013). The analytical tools (e.g. GIS, resource selection analysis, movement models) and information sources (e.g. satellite imagery, sea ice charts, polar bear location data) are available to refine polar bear harvest management and a precautionary approach that includes bear behavior, movement strategies, and sea ice habitat conditions would aid in conservation efforts.

## Supporting Information

**Table S1.**The number of clusters and individual bear cluster affiliations based on fuzzy clustering using fuzziness weighting exponent value (*m*) of 1.5 for the location data of satellite collared female polar bears, Foxe Basin, Nunavut, Canada, October–March 2007–2011. **Figure S1**. Fuzzy c-means validity functions plots: fuzziness performance index (FPI) and modified partition entropy (MPE) versus potential number of subpopulation clusters.
