# Supplementary material for: Population substructure and space use of Foxe Basin polar bears
Source: Ecol Evol. 2015 Jun 25;5(14):2851–64. doi: 10.1002/ece3.1571 (PMC4541990; doi:10.1002/ece3.1571)

Supplementary Material. Appendix S1.

Sahanatien, V., Peacock, E., and Derocher, A.E. 2015. Population substructure and space use of Foxe Basin polar bears. Ecology and Evolution

Table S1. The number of clusters and individual bear cluster affiliations based on fuzzy clustering using fuzziness weighting exponent value (m) of 1.5 for the location data of satellite collared female polar bears, Foxe Basin, Nunavut, Canada (October – March, 2007-2011). FB=Foxe Basin cluster, HS=Hudson Strait cluster and HB=Hudson Bay cluster. The table values indicate strength of a bear’s cluster affiliation and the highest values are bolded for each set of clusters.

|  | 2 Clusters | | 3 Clusters | | | 4 Clusters | | | |
| --- | --- | --- | --- | --- | --- | --- | --- | --- | --- |
| BearID | **FB/HS** | **HB/HS** | **FB** | **HS** | **HB** | **FB** | **HS** | **HB1** | **HB2** |
| B600661 | 0.0035 | **0.9965** | 0.0011 | 0.00161 | **0.99729** | 0.00147 | 0.00206 | 0.28817 | **0.70829** |
| B618529 | **0.99792** | 0.00208 | **0.95917** | 0.03769 | 0.00314 | **0.95365** | 0.03925 | 0.00447 | 0.00264 |
| B618532 | **0.99838** | 0.00162 | **0.98148** | 0.01646 | 0.00206 | **0.97728** | 0.01773 | 0.00316 | 0.00183 |
| B618535 | **0.90231** | 0.09769 | 0.00109 | **0.99856** | 0.00036 | 0.00137 | **0.99766** | 0.00048 | 0.00049 |
| B618537 | **0.97506** | 0.02494 | 0.0086 | **0.99041** | 0.00099 | 0.00617 | **0.99228** | 0.00083 | 0.00072 |
| A618542 | **0.99832** | 0.00168 | **0.9359** | 0.0603 | 0.0038 | **0.92849** | 0.06313 | 0.00511 | 0.00326 |
| A631643 | **0.97262** | 0.02738 | **0.98693** | 0.00991 | 0.00315 | **0.98407** | 0.00924 | 0.00434 | 0.00235 |
| A631681 | 0.00703 | **0.99297** | 0.00572 | 0.0101 | **0.98418** | 0.00335 | 0.00565 | **0.75822** | 0.23278 |
| A631682 | **0.52356** | 0.47644 | 0.17031 | **0.60576** | 0.22392 | 0.1413 | **0.46562** | 0.16263 | 0.23046 |
| A631684 | **0.98688** | 0.01312 | **0.7669** | 0.20218 | 0.03092 | **0.72578** | 0.2001 | 0.04756 | 0.02656 |
| A631687 | **0.89016** | 0.10984 | 0.09546 | **0.88357** | 0.02097 | 0.08385 | **0.87781** | 0.01928 | 0.01906 |
| A631688 | 0.23363 | **0.76637** | 0.03684 | **0.80378** | 0.15939 | 0.03439 | **0.66699** | 0.11833 | 0.18029 |
| A631691 | 0.35409 | **0.64591** | 0.02462 | **0.90856** | 0.06682 | 0.02563 | **0.83235** | 0.05717 | 0.08484 |
| A631692 | **0.99564** | 0.00436 | **0.97925** | 0.01707 | 0.00368 | **0.97085** | 0.01916 | 0.00658 | 0.00341 |
| A631694 | **0.96414** | 0.03586 | **0.98695** | 0.00892 | 0.00413 | **0.98143** | 0.00888 | 0.00635 | 0.00334 |
| A631695 | **0.88756** | 0.11244 | 0.02131 | **0.97265** | 0.00604 | 0.01776 | **0.9716** | 0.00543 | 0.00521 |
| A631716 | **0.96031** | 0.03969 | **0.73523** | 0.21466 | 0.05011 | **0.68288** | 0.20516 | 0.06785 | 0.0441 |
| A631718 | 0.01197 | **0.98803** | 0.00562 | 0.00759 | **0.98679** | 0.00403 | 0.00526 | **0.68591** | 0.3048 |
| A631720 | 0.0264 | **0.9736** | 0.01862 | 0.01219 | **0.96919** | 0.00127 | 0.00081 | **0.97774** | 0.02018 |
| B618532a | **0.99402** | 0.00598 | **0.99522** | 0.00413 | 0.00065 | **0.99494** | 0.00376 | 0.00081 | 0.00049 |
| B618535a | **0.88707** | 0.11293 | 0.00078 | **0.99894** | 0.00027 | 0.00072 | **0.99875** | 0.00027 | 0.00026 |
| B618537a | **0.98992** | 0.01008 | 0.06683 | **0.92909** | 0.00408 | 0.05571 | **0.93673** | 0.00428 | 0.00328 |
| A631643a | **0.9681** | 0.0319 | **0.95351** | 0.03776 | 0.00873 | **0.94546** | 0.0364 | 0.01111 | 0.00703 |
| A631684a | **0.88704** | 0.11296 | **0.72981** | 0.17337 | 0.09682 | **0.62619** | 0.15214 | 0.15343 | 0.06824 |
| A631692a | **0.99141** | 0.00859 | **0.97206** | 0.02223 | 0.00572 | **0.96028** | 0.02443 | 0.01014 | 0.00515 |
| A631694a | **0.90413** | 0.09587 | **0.95497** | 0.02525 | 0.01978 | **0.92984** | 0.02424 | 0.03115 | 0.01477 |
| A617098 | 0.00601 | **0.99399** | 0.00312 | 0.00476 | **0.99212** | 0.0025 | 0.00365 | 0.22397 | **0.76988** |
| A618527 | 0.03138 | **0.96862** | 0.02057 | 0.01458 | **0.96485** | 0.00245 | 0.00169 | **0.95684** | 0.03903 |
| A618536 | **0.95891** | 0.04109 | **0.97262** | 0.0202 | 0.00718 | **0.96558** | 0.0192 | 0.00961 | 0.00561 |
| A618542a | **0.99612** | 0.00388 | **0.80354** | 0.18593 | 0.01052 | **0.7838** | 0.19316 | 0.01371 | 0.00933 |
| A34046 | 0.00476 | **0.99524** | 0.00225 | 0.00389 | **0.99387** | 0.00056 | 0.00092 | 0.03345 | **0.96508** |
| A34051 | **0.83502** | 0.16498 | 0.1269 | **0.82673** | 0.04638 | 0.12138 | **0.78403** | 0.04398 | 0.05061 |
| A34056 | 0.00587 | **0.99413** | 0.00497 | 0.01266 | **0.98237** | 0.00121 | 0.00292 | 0.04785 | **0.94802** |
| A34058 | 0.00284 | **0.99716** | 0.00212 | 0.0049 | **0.99297** | 0.00054 | 0.00118 | 0.03529 | **0.96299** |
| A77012 | 0.00805 | **0.99195** | 0.00497 | 0.00717 | **0.98786** | 0.00279 | 0.00385 | 0.16094 | **0.83242** |

Fig. S1. Fuzzy c-means validity functions: fuzziness performance index (FPI) and modified partition entropy (MPE) vs potential number of subpopulation clusters. The diagonal metric distance was used in the calculations along with fuzzy weighting exponent (m) 1.5≤ m ≤ 3.0 for satellite collared female polar bears, Foxe Basin, Nunavut, Canada (October-March 2007-2011). The purpose of the plots is to identify the FPI and MPE minima as a means to determine movement behaviour: independent or coordinated. The minima for each exponent value (m) occurred at 34 clusters which is equal to the total sample size, thus each polar bear moved independently of all other bears.





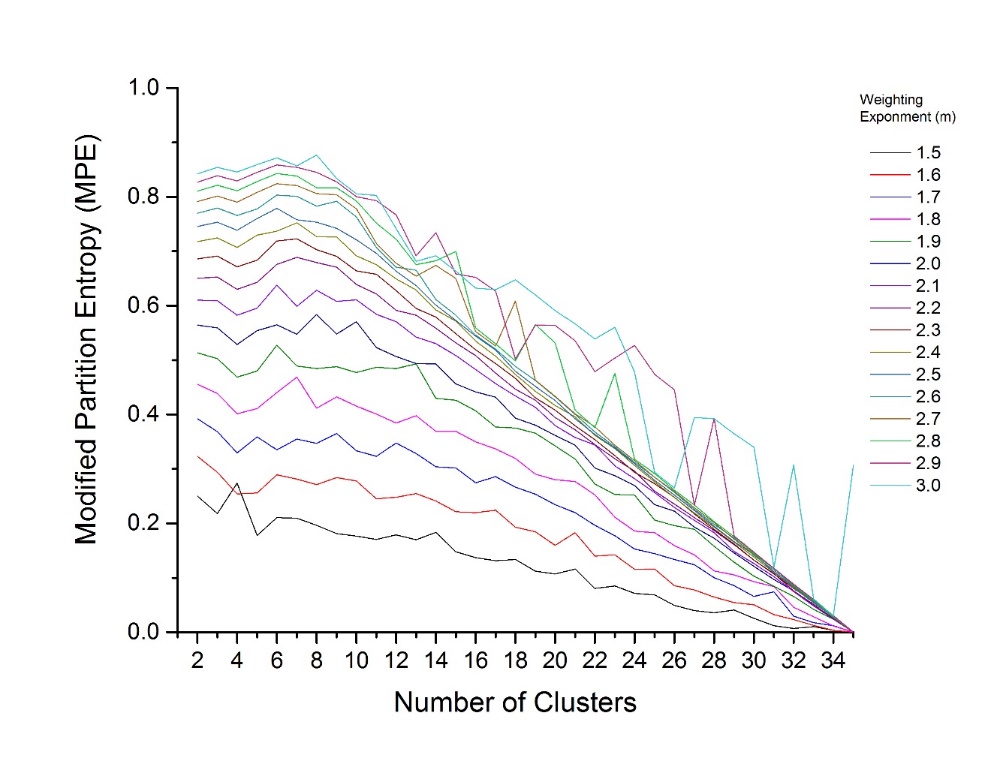

Supplement: Supplementary file 1 [file ece30005-2851-sd1.docx]
